# Effect of Synthetic Vitreous Fiber Exposure on TMEM16A Channels in a *Xenopus laevis* Oocyte Model

**DOI:** 10.3390/ijms25168661

**Published:** 2024-08-08

**Authors:** Martina Zangari, Giuliano Zabucchi, Martina Conti, Paola Lorenzon, Violetta Borelli, Andrew Constanti, Francesco Dellisanti, Sara Leone, Lisa Vaccari, Annalisa Bernareggi

**Affiliations:** 1 Department of Life Sciences, University of Trieste, 34127 Trieste, Italy; martina.zangari@unibo.it (M.Z.); plorenzon@units.it (P.L.); borelliv@units.it (V.B.); 2 CERIC-ERIC, Strada Statale 14, Km 163.5, AREA Science Park, 34149 Trieste, Italy; 3 Elettra-Sincrotrone Trieste S.C.p.A., Strada Statale 14, Km 163.5, AREA Science Park, Basovizza, 34149 Trieste, Italy; lisa.vaccari@elettra.eu; 4 CNR-IOM—Istituto Officina dei Materiali, Consiglio Nazionale delle Ricerche, Area Science Park, Basovizza, Strada Statale 14, Km 163.5, 34149 Trieste, Italy; conti@iom.cnr.it; 5 Department of Pharmacology, UCL School of Pharmacy, London WC1N 1AX, UK; a.constanti@ucl.ac.uk; 6 ANALITICA—Mineralogical and Envirnomental Laboratory, San Lazzaro di Savena, 40068 Bologna, Italy; f.dellisanti@analitica-lab.it; 7 S.C. Prevenzione e Sicurezza negli Ambienti di Lavoro Laboratorio Fibre, ASUGI—Azienda Sanitaria Universitaria Giuliano Isontina, Via Sai, 34128 Trieste, Italy; sara.leone@asugi.sanita.fvg.it

**Keywords:** TMEM16A channels, synthetic vitreous fibers, asbestos fibers, *Xenopus laevis* oocytes, voltage clamp

## Abstract

Many years ago, asbestos fibers were banned and replaced by synthetic vitreous fibers because of their carcinogenicity. However, the toxicity of the latter fibers is still under debate, especially when it concerns the early fiber interactions with biological cell membranes. Here, we aimed to investigate the effects of a synthetic vitreous fiber named FAV173 on the *Xenopus laevis* oocyte membrane, the cell model we have already used to characterize the effect of crocidolite asbestos fiber exposure. Using an electrophysiological approach, we found that, similarly to crocidolite asbestos, FAV173 was able to stimulate a chloride outward current evoked by step membrane depolarizations, that was blocked by the potent and specific TMEM16A channel antagonist Ani9. Exposure to FAV173 fibers also altered the oocyte cell membrane microvilli morphology similarly to crocidolite fibers, most likely as a consequence of the TMEM16A protein interaction with actin. However, FAV173 only partially mimicked the crocidolite fibers effects, even at higher fiber suspension concentrations. As expected, the crocidolite fibers’ effect was more similar to that induced by the co-treatment with (Fe^3+^ + H_2_O_2_), since the iron content of asbestos fibers is known to trigger reactive oxygen species (ROS) production. Taken together, our findings suggest that FAV173 may be less harmful that crocidolite but not ineffective in altering cell membrane properties.

## 1. Introduction

In 1987, the International Agency for Research on Cancer (IARC) classified asbestos fibers as carcinogenic to humans (Group 1) and, since then, the production, import, and trade of asbestos was banned in the whole of Europe [1]. Synthetic vitreous fibers, also known as man-made vitreous fibers (MMVFs), have replaced asbestos because of their low cost and heat resistance, but also because they are supposed to be environmentally safer. MMVFs are commonly used for thermal and sound insulation purposes, or for gasket and filter production [2]. They are divided into wools (refractory slang wool, rock wool, glass wool, and ceramic fibers), made of a mass of tangled, discontinuous fibers of variable lengths and diameters, and filaments (continuous glass filaments) made of filaments of continuous uniform fibers [2]. They are synthetically made by spraying or extruding molten glass, rock, or furnace slag in a melting combination of several compounds, mainly alumina (Al_2_O_3_) and silicon (SiO_2_), but also oxides of alkaline earths, alkalis, boron, iron, and zirconium [3]. Their chemical composition is quite variable, even within the same categories; for instance, the rock wool contains SiO_2_ and Al_2_O_3_ in the ranges of 43–50% and 6–15% (*w*/*w*), respectively, while, in glass wool, the ranges are 55–70% and 0–7% (*w*/*w*), respectively. They share an amorphous structure with glass, since the material is cooled in an amorphous liquid state at a rate too fast to form a crystal [2]. 

Even though their use dates back many years, the effects of MMVF exposure on human health are still a matter of debate. In 2001, the IARC classified the glass and rock wools into Group 2B, and the glass filaments into Group 3. The limited carcinogenicity risk was associated with the MMFV bio-solubility, which is favored by the amorphous structure [4], by the percentages of CaO, MgO, Na_2_O, B_2_O_3_, and BaO [5], and by the ratio of Al/Si [6]. More recently, in 2023, Egnot and coworkers, reviewing 23 studies, excluded any association between occupational MMVF exposure and non-malignant respiratory disease. However, the evidence was based on old studies (over 20 years ago), and it was supported by a poor statistical analysis [7,8].

If the evaluation of MMVF toxicity is still unclear, this does not apply for asbestos fibers [9]. Asbestos fibers are divided into two mineral groups that differ in composition and structure: the serpentines and the amphiboles. The first contains a single asbestiform variety named chrysotile, while the amphiboles include anthophyllite, amosite, crocidolite, tremolite, and actinolite, and they are richer in iron [10]. Among the mechanisms suggested to be responsible for the asbestos-correlated diseases, the fiber iron content and ROS production are those specifically related to crocidolite (Croc) exposure [11]; this amphibole generates the highest amount of ROS compared to other asbestos fibers, as a consequence of the mobilizable surface iron that catalyzes the formation of hydroxyl radicals by either the Fenton or Haber–Weiss reactions [5]. Rock and glass wools are considered to be less harmful with respect to asbestos, but may not entirely be without hazard. Early studies (1991–2015) showed that the latter fibers, mainly amphiboles, contain and release a high percentage of iron (II), responsible for iron-mediated ROS production, which exerts cytotoxic and genotoxic damage [12,13]. Conversely, MMVFs and rock ceramic fibers (RCFs) exert a small genotoxic effect, which was shown to be iron-independent. Despite this, MMVFs and RCFs can release iron in amounts comparable to that of amphiboles. It has been proposed that it is the iron that remains fiber-associated (high in amphiboles and low in MMVFs), which is the most important, and not the released amount [14]. The genotoxic effect of MMVFs has been confirmed by studies in animal models reviewed in Enterline (1991) [15]. These studies revealed that glass fibers and RCFs are carcinogenic in hamsters, more so than in rats, when given directly intraperitoneally. Their carcinogenic power is however weaker than that of asbestos [15]. These seminal findings suggest that either the animal species or the extent of exposure (which is at a very high level when directly given) is important for determining the pathogenicity of MMVFs. The risk for human beings is moderate, but, again, its degree may be dependent on the extent of exposure. 

The direct cytotoxic effect of MMVFs has been considered by many authors. It was measured as lactate dehydrogenase (LDH) release, hemolysis, MTT reduction, and ROS production in rat alveolar macrophages, human mesothelial cells (MET5A), and alveolar epithelial cells (A549) [14,16,17]. These findings suggest that MMVFs exert a weak activity with respect to asbestos, and are able to trigger ROS production from neutrophils [17]. Taken together, the results obtained suggest that MMVFs have the capacity of inducing cell injury, even if weaker with respect to asbestos fibers, and this capacity may depend on the extent of exposure. This being the case, the cytotoxicity of MMVFs deserves to be investigated in more detail and compared with that of asbestos fibers.

In line with this, the treatment of *Xenopus laevis* oocytes, with Croc fiber suspensions, was found to stimulate the release of H_2_O_2_, and the co-treatment with exogenous Fe^3+^ + H_2_O_2_ altered the electrical membrane properties of the oocytes similarly to that observed with Croc [18]. In both cases, the alterations were largely mediated by the activation of TMEM16A Ca^2+^-activated Cl^−^ channels [19], natively expressed in *Xenopus* oocytes [20,21]. In these cells, the overexpression of the protein also affected the cell morphology [22], while, in mammalian cells, the overexpression is associated with the development of tumors similar to those induced by asbestos fiber exposure [23]. For this reason, TMEM16A is becoming an intriguing key player in the early interactions of asbestos with the cell membrane, and may represent one of the first targets interacting with asbestos fibers [24,25,26]. In light of this, we wondered if the interaction of MMVFs with the cell membrane could also involve this membrane channel. 

Using *Xenopus* oocytes as a cell model, we analyzed the effects of MMFV exposure on electrical membrane properties, and the possible involvement of the TMEM16A channels. In addition, we compared the consequences of such an exposure at the morphological level, with those evocated by Croc and Fe^3+^ + H_2_O_2_.

## 2. Results

### 2.1. Effects of FAV173 Fiber Exposure on the Passive Membrane Properties of Xenopus Oocytes

**Figure 1:**
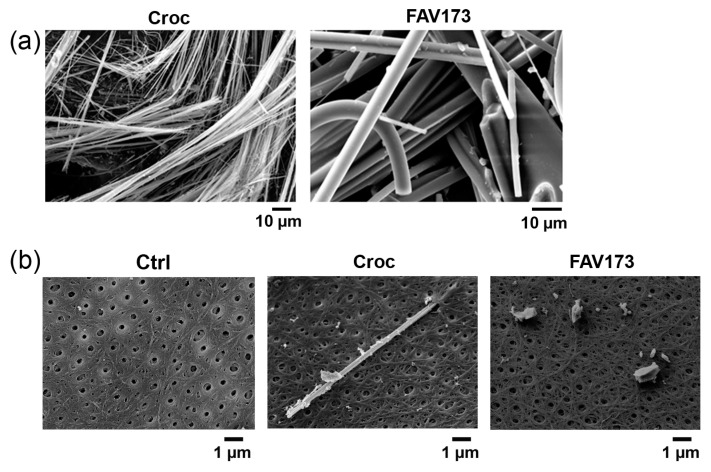
(**a**) Example of SEM images of Croc and FAV173 fibers. (**b**) Vitelline membrane of non-treated (Ctrl), Croc-treated, and ground FAV173-treated oocytes. Note, in (**b**), a Croc fiber partially inserted into the pore of the vitelline membrane. Croc = 15 μg/mL, FAV173 = 200 μg/mL.

Figure 1 shows representative SEM images of Croc and FAV173 fibers (Figure 1a), and on the top of the vitelline membrane of the oocytes (Figure 1b). As can be seen in Figure 1b, the vitelline envelope of the *Xenopus* oocyte is made of an interlacing network of small filaments, below which there are microvilli and the plasma membrane. Table S1 of Supplementary Materials summarizes the composition of the two types of fibers; note that the percentage of iron in Croc is almost 38% in weight, while that in FAV173 fibers is only 10.7%. 

**Figure 2:**
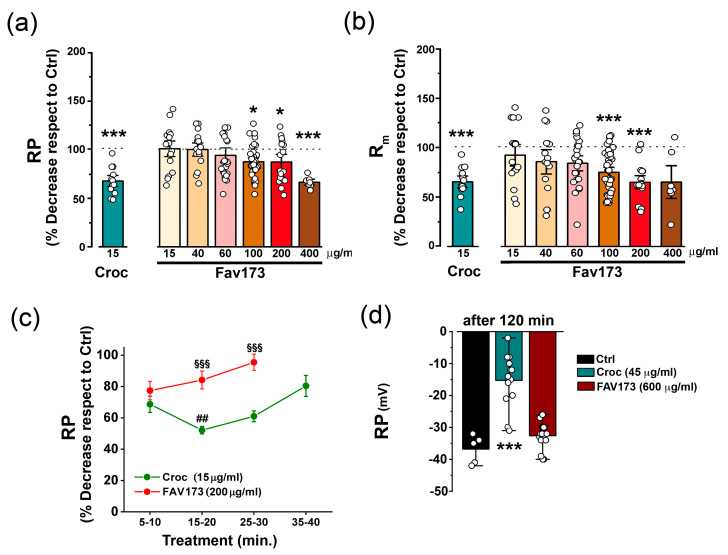
Dose–response effect of FAV173 fiber exposure (from 15 to 400 μg/mL) on resting potential (RP) in (**a**) and membrane resistance (R_m_) in (**b**). In (**a**,**b**), the green bars show the effect of 15 μg/mL of Croc for comparison. The effect of FAV173 and Croc is expressed as % of decrease with respect to untreated cells (Ctrl) from the same donor. * *p* < 0.05, *** *p* < 0.001 fibers vs. Ctrl (unpaired *t*-test). In (**c**), the time course effect of Croc and FAV treatments on RP (*n* ≥ 3 donors). ^§§§^ *p* < 0.001 FAV173 vs. Croc, ^##^ *p* < 0.01 Croc (15–20) vs. Croc (5–10), (unpaired *t*-test). In (**d**), the effect on RP was irreversible only at higher fiber concentrations in Croc-treated cells (120 min, Croc: 45 μg/mL; FAV173: 600 μg/mL). *** *p* < 0.001 Croc vs. Ctrl (one-way ANOVA with Tukey’s post hoc).

The effect of FAV173 fiber suspension exposure (from 15 μg/mL up to 400 μg/mL) on the *Xenopus* oocyte membrane was initially evaluated by measuring the resting membrane potential (RP) and membrane input resistance (R_m_) values following 5–40 min of treatment. Figure 2a,b show the dose–response relationship of the treatment. Note that, in the same figures, the effect of the exposure to a Croc fiber suspension (15 μg/mL; 5–40 min, *n* = 3 batches) is also included for comparison. 

In line with our previous findings [27], the asbestos treatment decreased the RP to 67 ± 4% of that in Ctrl cells (Figure 2a, e.g., in one batch, Ctrl: −44.62 ± 1.97 mV, *n* = 16; Croc: −29.54 ± 1.73 mV, *n* = 13; *** *p* < 0.001, not shown) and reduced the R_m_ to 65 ± 4% of the Ctrl value (Figure 2b, e.g., in one batch, Ctrl: 0.79 ± 0.03 MΩ, *n* = 16; Croc: 0.51 ± 0.04 MΩ, *n* = 13; ** *p* < 0.01, not shown). Interestingly, FAV173 elicited similar effects at a concentration of 400 μg/mL (RP decreased to 66 ± 4% of Ctrl cells; Figure 2a, e.g., in one batch, Ctrl: −46.60 ± 5.35 mV, *n* = 4; FAV173: −31.14 ± 1.30 mV, *n* = 7; ** *p* < 0.01, no shown) and 200 μg/mL, respectively (Figure 2b, e.g., in one batch, Ctrl: 0.91 ± 0.07 MΩ, *n* = 17; FAV173: 0.59 ± 0.04 MΩ, *n* = 18; *** *p* < 0.001). 

Both Croc and FAV173 fibers displayed a “time-window” effect on RP but with a different duration of action. Their effect was detected after 5–10 min of treatment, but, in FAV173-treated cells, the effect was shorter (Figure 2c). Moreover, while, by increasing the Croc fiber suspension concentration to 45 μg/mL, the Croc effect on RP was still present after 2 h (Ctrl: −36.98 ± 1.31 mV, *n* = 5; Croc: −15.27 ± 2.18 mV, *n* = 15; *** *p* < 0.001 vs. Ctrl, same donor), the FAV173 effect on RP remained transient even at a concentration of 600 μg/mL (FAV173: −32.60 ± 1.11 mV, *n* = 15, same donor; Figure 2c). Taken together, the results suggested that, even at higher concentrations, FAV173 was able to mimic only partially the most severe Croc effects on the oocyte membrane.

### 2.2. FAV173 Fiber Exposure Stimulates TMEM16A Channel Activity 

As previously mentioned, the alteration of the *Xenopus* oocyte membrane permeability following Croc exposure was largely attributed to the activation of TMEM16A channels endogenously expressed in the oocyte membrane [19]. Notably, a similar alteration was observed in human cell line A549 (Figure S1 of Supplementary Materials). These TMEM16A channels are activated by stepping the membrane potential from −80 mV to +40 mV (3 s, V_h_ = −40 mV) under a voltage clamp. They are permeable to Cl^−^ and are sensitive to [Ca^2+^]_i_ [28].

**Figure 3:**
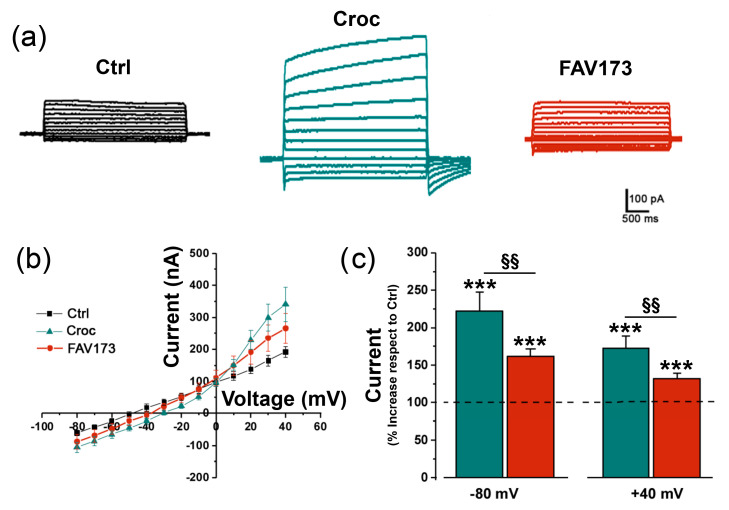
In (**a**), examples of current traces recorded by stepping the membrane potential from −80 to +40 mV (10 mV steps, 3 s, V_h_ = −40 mV) in non-treated (Ctrl, black), Croc-treated (green), and FAV173-treated (red) cells. (**b**) I–V relationships from Ctrl, Croc-treated, and FAV173-treated cells (same batch). Note the outward rectification in treated cells. (**c**) Percentage increase in the evoked current amplitude measured at −80 mV and +40 mV and normalized to their respective Ctrl (of the same donor). *** *p* < 0.001, Fiber-treatments vs. Ctrl; ^§§^ *p* < 0.001, Croc vs. FAV173 (unpaired *t*-test). Croc: 15 μg/mL; FAV173: 200 μg/mL.

Figure 3a shows examples of current traces recorded in Ctrl, Croc-treated (15 μg/mL), and FAV173-treated cells (200 μg/mL), respectively. Both in the presence of Croc and FAV173, the I–V relationships (Figure 3b, Ctrl: *n* = 6; Croc: *n* = 9, FAV173: *n* = 5) revealed an outward rectification typical of the TMEM16A current activation (Figure 3b). Moreover, both treatments increased the amplitude of the evoked currents with respect to the Ctrl condition (Figure 3c). However, the Croc effect was stronger when compared to FAV173 (at −80 mV: Croc: 222 ± 25%; FAV173: 162 ± 10%, ^§§^ *p* < 0.01; at +40 mV Croc: 173 ± 17%; FAV173: 132 ± 7%, ^§§^ *p* < 0.01, Croc: *n* = 32, 5 batches, FAV173: *n* = 53, 7 batches; Figure 3c).

**Figure 4:**
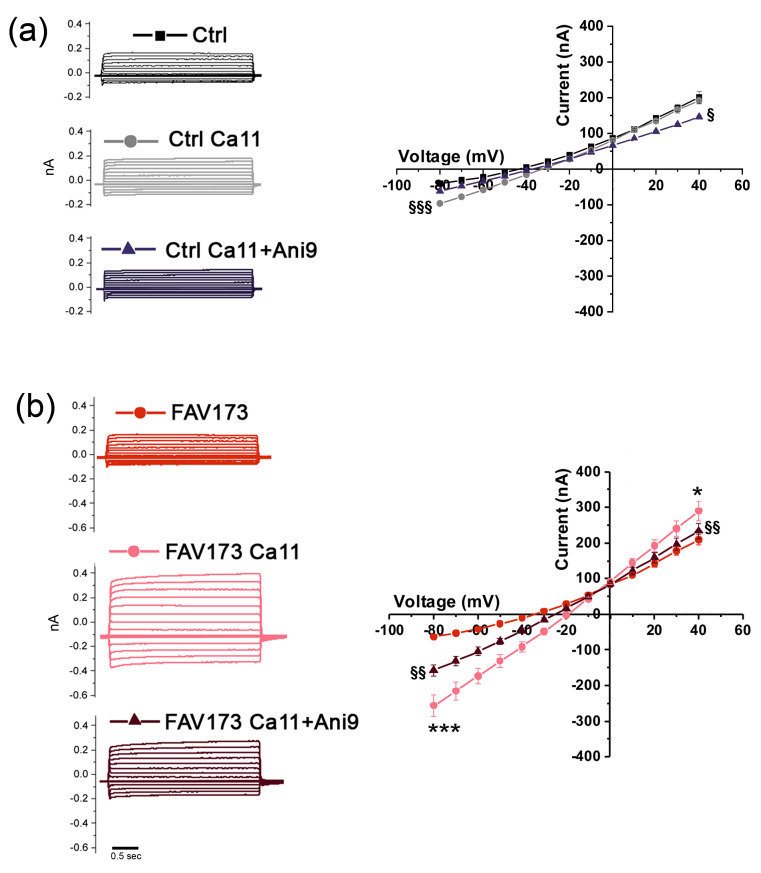
(**a**, **left**) Examples of recording traces from Ctrl cells in normal bathing solution, in presence of [Ca^2+^]_e_ = 11 mM (Ca11) and Ca11 + Ani9 (1 μM). (**a**, **right**) I–V relationships in Ctrl (normal bathing solution), Ca11, and Ca11 + Ani9 (1 μM) conditions. Note that, in Ca11, the currents at negative potentials increased with respect to those recorded in normal bathing solution, and the evident blocking effect of Ani9. (**b**, **left**) Evoked currents from FAV173-treated (200 μg/mL) cells in presence of Ca11 and Ca11 + Ani9 (1 μM). (**b**, **right**) The I–V relationships revealed a significant increase in the evoked currents at positive and negative potentials in presence of Ca11 and a partial blocking effect induced by Ani9. Ctrl: *n* = 5; FAV173: *n* = 7, same donor. * *p* < 0.05, *** *p* < 0.001, Fibers vs. Ctrl (unpaired *t*-test); ^§^ *p* < 0.05, ^§§^ *p* < 0.01, ^§§§^ *p* < 0.001, Ca11 vs. Ca11 + Ani9 (paired *t*-test).

The results indicated that the TMEM16A channels are possible mediators of the FAV173 effect, as well as of the Croc effect. To further investigate such a possibility, we took advantage of the [Ca^2+^]_i_ sensitivity of TMEM16A and the availability of Ani9, a potent and selective TMEM16A channel blocker. Preparatory experiments conducted on Ctrl cells under the Ca11 bathing condition showed a slight increase in the evoked current amplitudes with respect to those recorded in the normal bathing solution. The increase in amplitude was abolished in the presence of the blocker Ani9, confirming the presence of endogenous TMEM16A channels in *Xenopus* oocytes and the enhancing effect of Ca11 on their activity (−80 mV, Ca11 = −96.20 ± 5.21 nA, Ca11 + Ani9: –61.60 ± 8.95 nA, ^§§§^ *p* < 0.001; +40 mV Ca11 = 191.80 ± 10.08 nA, Ca11 + Ani9: 146.20 ± 4.76 nA, ^§^ *p* < 0.05, *n* = 5; Figure 4a). We then recorded the “FAV173-induced currents” under Ca11 conditions and after the application of the selective blocker Ani9 (1 μM, [29]). In FAV173-treated oocytes (*n* = 7), Ca11 increased the evoked current amplitudes recorded at both positive and negative potentials with respect to the normal bath solution (−80 mV, FAV173 = −63.83 ± 4.07 nA, FAV173 in Ca11: −257.10 ± 30.60 nA, *** *p* < 0.001; +40 mV, FAV173 = 209.30 ± 13.46 nA, FAV173 in Ca11: 289.60 ± 27.82 nA, * p < 0.05; Figure 4b). Moreover, Ani9 was found to partially reverse the fibers’ effect (e.g., −80 mV, Ca11 + Ani9: 157.90 ± 15.77 nA, ^§§^ *p* < 0.001 vs. FAV173 in Ca11; +40 mV, Ca11 + Ani9: 233.70 ± 21.32 nA, ^§§^ *p* < 0.04 vs. FAV173 in Ca11; Figure 4b). This evidence strongly suggested the contribution of TMEM16A channel activity to the FAV173-mediated effect on the oocyte cell membrane. 

### 2.3. Effects of FAV173 Fiber Exposure on Cell Morphology 

In Xenopus oocytes, TMEM16A controls the electrical properties of the cell membrane but also the morphology of the microvilli [22]. Therefore, a set of experiments was carried out to explore the effects on microvilli morphology 120 min after the following treatments: 200 μg/mL of FAV173 in the Ca11 bathing condition (to enhance the effect on TMEM16A channel activation), 15 μg/mL of Croc, and 400 μM Fe^3+^ + 1 mM H_2_O_2_, the latter to mimic the iron content and ROS production by the fibers, as reported in detail in our previous study [18]. 

**Figure 5:**
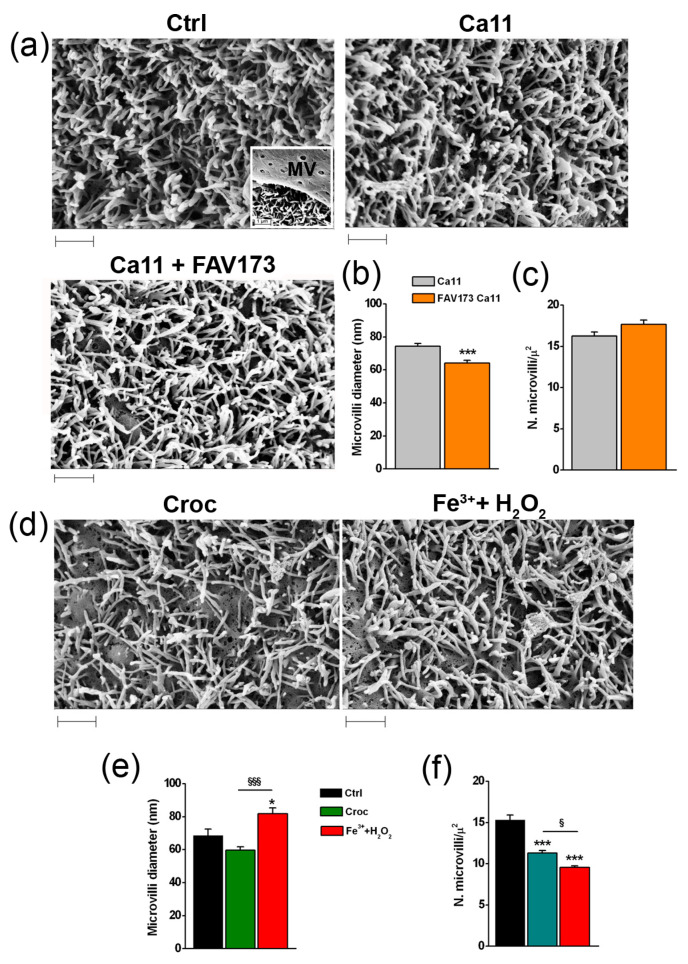
Example of SEM images of microvilli in (**a**) Ctrl oocyte, and oocytes in Ca11 bath condition in the absence and in the presence of FAV173 (200 μg/mL). In the inset of Ctrl, the vitelline membrane (VM) above the microvilli (MV) is shown. The FAV173 effect on the diameter (**b**) and density (**c**) of the microvilli (**c**), *** *p* < 0.001 vs. Ca11 (unpaired *t*-test). In (**d**) microvilli of Croc and (Fe^3+^ + H_2_O_2_)-treated oocytes. Comparison of microvilli diameter (**e**) and density (**f**) values measured under the 3 test conditions (* *p* < 0.05, *** *p* < 0.001 vs. Ctrl, ^§^ *p* < 0.05, ^§§§^ *p* < 0.001 (Fe^3+^ + H_2_O_2_) vs. Croc, One-Way ANOVA with Tukey’s post hoc). Cells were treated for 60 min. (values are in the text). Scale bar 1 μm.

The microvilli became clearly visible when the covering vitelline membrane (MV) of the oocyte was removed (Figure 5a). In the Ca11 bathing condition, the surface morphology of the cells remained roughly unchanged with respect to that seen in the normal Ca11 bathing solution (Figure 5a, Ctrl vs. Ca11) excluding any alteration due to the high [Ca^2+^]_e_ per se. The treatment with FAV173 reduced the microvilli diameter with respect to Ctrl cells (from 74.32 ± 1.68 nm to 64.06 ± 1.80 nm, *** *p* < 0.001, *n* = 104 and *n* = 105, respectively; Figure 5b), leaving unaltered their density (Figure 5c). After Croc treatment in the normal bathing condition (Figure 5d), the microvilli diameter decreased (Ctrl: 68.50 ± 3.96 nm, *n* = 26; Croc: 59.56 ± 2.14 nm, *n* = 37; *p* = 0.06; Figure 5e), as well as the density (from 15.31 ± 0.58, *n* = 14 to 11.32 ± 0.30, *n* = 14 microvilli per μm^2^; *** *p* < 0.001; Figure 5f). By comparison, the co-treatment with Fe^3+^ + H_2_O_2_ increased diameter (Fe^3+^ + H_2_O_2_: 81.83 ± 3.41 nm, *n* = 66, * *p* < 0.001 vs. Ctrl, ^§§§^ *p* < 0.001 vs. Croc; Figure 5e) and reduced the microvilli density (9.58 ± 0.18 microvilli per μm^2^ *n* = 12, *** *p* < 0.001 vs. Ctrl, ^§^ *p* < 0.05 vs. Croc; Figure 5f). 

The lack of FAV173 effect on the microvilli density thus seemed to exclude the involvement of Fe^3+^ and H_2_O_2_ in the development of any FAV173-induced morphological effect. On the contrary, the iron content and ROS-induced production could contribute to the effects observed after Croc exposure.

### 2.4. The Role of Actin in the TMEM16A-Mediated Effect Induced by FAV173 Fiber Exposure

**Figure 6:**
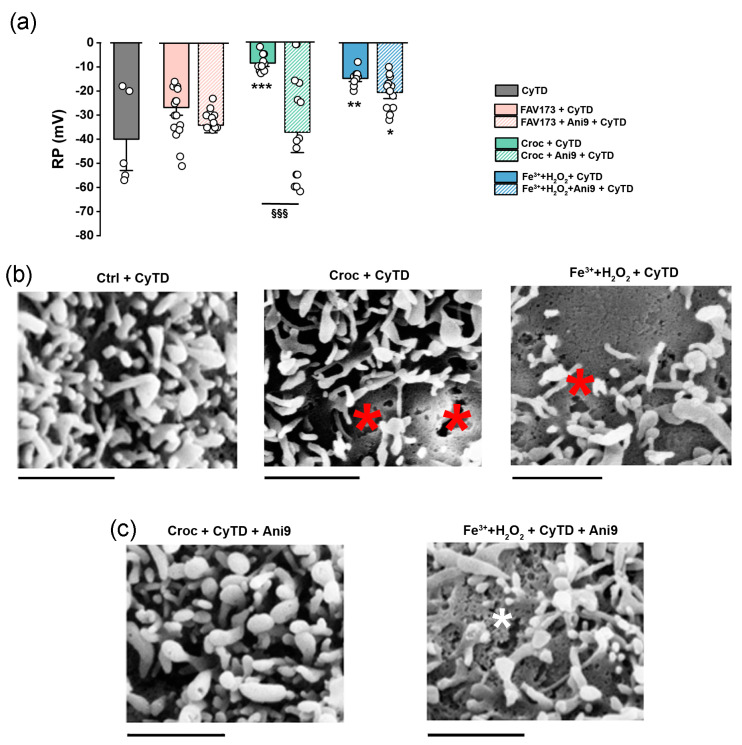
(**a**) Comparison of RP values in oocytes in the presence of CyTD alone (*n* = 5), or with FAV173 (*n* = 13) and Ani9 (*n* = 13), Croc (*n* = 12) and Ani9 (*n* = 15), (Fe^3+^ + H_2_O_2_) (*n* = 12), and Ani9 (*n* = 14). * *p* < 0.05, ** *p* < 0.01, *** *p* < 0.001 vs. CyTD, ^§§§^ *p* < 0.001 CyTD + Croc vs. CyTD + Croc + Ani9 (ANOVA with Tukey’s post hoc). (**b**) Examples of SEM images of the plasmalemma from CyTD, CyTD + Croc, and (Fe^3+^ + H_2_O_2_) + CyTD-treated oocytes. In both cases, damage on the plasmalemma are visible (red asterisks). In (**c**), the presence of Ani9 fully recovered the membrane damage in the Croc-treated cells, while it is still visible in (Fe^3+^ + H_2_O_2_)-treated cells (white asterisk). Scale bar: 1 μm.

TMEM16A is physically associated with ezrin–radixin–moesin (ERM) to create a cross-link between the oocyte plasma membrane and the cytoskeleton complex [22]. This type of interaction organizes the cortical cytoskeleton by linking actin to the plasma membrane to coordinate cell signalling events by scaffolding molecules in many cell types [30]. To compare the consequence of such an interaction under the fibers’ exposure, we blocked the actin polymerization with cytochalasin D (CyTD). In the control condition, after 120 min of CyTD, the mean RP of the oocyte cells was −40.00 ± 8.65 mV (*n* = 5, CyTD, 5 μM), and FAV173 only slightly depolarized the RP of CyTD-treated cells; in this case, the RP was −26.69 ± 2.12 mV (*n* = 13; Figure 6a), similar to that in Ani9 (−30.00 ± 2.12 mV, *n* = 13). On the other hand, in Croc, the mean RP was depolarized to −8.75 ± 0.95 mV (*n* = 12, 120 min of co-treatment, ** *p* < 0.001 vs. CyTD). When Ani9 was added at the beginning of the treatment, the RP values remained similar to that in CyTD alone (−37.4 ± 5.64 mV, *n* = 15, ^§§§^ *p* < 0.001, CyTD + Croc vs. CyTD + Croc + Ani9; Figure 6a), thus preventing the Croc effect. Lastly, we compared the effect of (Fe^3+^ + H_2_O_2_) on CyTD-treated cells, as also summarized in Figure 6a. The oocytes behaved similarly to those treated with Croc, showing a mean RP value of −14.75 ± 0.89 mV (*n* = 12), but, here, Ani9 only partially recovered the effect (−20.57 ± 1.73 mV, *n* = 14). The partial recovery under the Ani9 treatment was in line with the plasmalemma damage still visible only in the latter case (see asterisks of Figure 6b,c).

## 3. Discussion

Having previously found that asbestos Croc fibers affect the oocyte electrical membrane properties by altering the TMEM16A channel activity in the *Xenopus* oocyte, here, we evaluated whether the synthetic vitreous fiber named FAV173 was similarly able to affect the cell membrane properties in the same cell model [27]. 

A limitation of the oocyte model is the difficulty of studying the cytoplasmic ultrastructure following the fiber interaction: the cytoplasm of these cells is rich in strongly electrodense granules, which completely mask the other subcellular organelles, and the visibility of the fibers inside the cell. Thus, the use of human cells is ideally more desirable in order to further analyze the intracellular mechanisms induced by the fiber exposure. On the other hand, Xenopus oocytes are a more convenient and powerful tool to investigate the changes in the electrical membrane properties as a result of their size, and the ease with which classical electrophysiological techniques can be used, as largely reported in the literature [31]. This was confirmed by the results of the set of experiments performed in the human lung cancer cell line A549 exposed to Croc (Figure S1 of Supplementary Materials), with results in line with those observed in amphibian oocytes. Moreover, the expression of TMEM16A channels is well-documented in Xenopus oocytes [19,20,21], as well as in lung cells [32,33].

Our results revealed that only above 100 μg/mL suspension concentrations did the FAV173 fibers induce changes in the passive membrane properties (RP and R_m_) similar to those elicited by Croc fiber exposure at a lower concentration. Moreover, even though the electrical effects were transitory for both types of fibers, in the case of Croc exposure, they lasted longer and became irreversible at higher concentrations, suggesting that asbestos fibers were more harmful for the cells than the synthetic vitreous counterparts. Note that, if the concentration of Croc is in the range of that used in cell biology studies on asbestos effects [34], it is still higher with respect to that accumulated in the lungs of exposed human beings [35]. Conversely, the concentration employed for studying the FAV173 effect is comparable to that employed in animal models where the effect of other particles was studied [36]. 

As reported elsewhere [18,19], Croc exposure increased the amplitude of evoked (stimulated) outward currents, which displayed an outward rectification similar to that reported for TMEM16A channels. To a lesser extent, a similar effect was detected in the presence of FAV173 fibers. Moreover, in high [Ca^2+^]_e_ (Ca11 bathing condition), the FAV173-induced currents increased dramatically in amplitude and were blocked by the specific TMEM16A channel antagonist Ani9, indicating that a Ca^2+^-dependent activation of TMEM16A currents also contributed to the FAV173-mediated effect. FAV173 as well as Croc treatment altered the oocyte microvilli morphology, and such an effect was detectable 120 min after exposure, i.e., when the electrical membrane properties were fully recovered (within 60 min). However, while FAV173 (in the Ca11 condition) only reduced the microvilli diameter, Croc also decreased their density. Albeit still speculative, the reason why FAV173 fibers did not affect the microvilli morphology similarly to Croc fibers could be attributed to a different content of iron (e.g., Fe_2_O_3_: 10.7% vs. 26.5% of Croc) and, thus, a specific greater capability of Croc fibers to stimulate ROS production. In accordance with this hypothesis, we observed a similar decreased microvilli density after the combined treatment with Fe^3+^ + H_2_O_2_. If this was the case, both fibers (FAV173 and Croc) could share the initial and transitory mechanism of action at the membrane level, during which the TMEM16A channel activation could be a consequence of the direct fiber interaction with the plasmalemma [18,19]. It has been reported that, when the cells are challenged with the same fiber concentrations, Croc fibers appear free in the cytosol, while the synthetic vitreous FAV173 fibers are invariably engulfed in classical phagosomes [37] making unlikely the possibility of a piercing membrane process for FAV173 fibers. However, in our experiments, we employed FAV173 fibers at a high suspension concentration to mimic the effect of Croc fibers; thus, we cannot exclude such a possibility. Thus, we propose that, under our experimental conditions, FAV173 fibers could promote a Ca^2+^ influx by piercing the cell membrane, that would then activate the TMEM16A channels as reported in the case of the Croc fibers [19]. 

On the other hand, the possibility of piercing the cell membrane at very high FAV173 concentrations (higher than 100 μg/mL) is only based on electrophysiological observations, with respect to what was observed with Croc fibers. While we cannot exclude that a membrane piercing really occurs, its occurrence will not change our conclusion. The high concentration employed had no real biological/clinical significance. It simply confirms that FAV173 fails to modify the electrophysiology of the oocyte membrane at low concentrations, where Croc already shows its maximal effect.

The transient effects of Croc on the oocyte cell membrane are likely due to alterations in the cytoskeleton quickly repaired by peripheral actin contraction [18]: by blocking the actin polymerization with CyTD, the RP of Croc- and (Fe^3+^ + H_2_O_2_)-treated cells (but not that of FAV173-treated cells) remained irreversibly depolarized because the membrane lesions most likely became permanent. Specifically, a long-lasting fiber treatment with CyTD caused a loss of RP in Croc- and (Fe^3+^ + H_2_O_2_)-treated cells (likely dead cells [38]), while FAV-treated oocytes were not affected, revealing that the latter fiber cannot severely interact with oocytes. Interestingly, the fact that Ani9 could completely prevent the Croc effect underlines once more the role of the TMEM16A protein in the Croc-mediated effect at the cell membrane level. This finding suggests a new method for the screening of the MMVF toxicity CyTD-induced irreversibility: in its absence, as is the case with FAV173, it is very likely that the fibers will be considered devoid of any toxic capacity.

In conclusion, the electrophysiological modification induced by FAV173 fibers in *Xenopus* oocytes appears to be dependent on the TMEM16A channel activation similarly to Croc fibers, even if FAV173 fibers show a less severe effect. The present study, thus, underlines the key role of TMEM16A channels in the development of the effects of silicate fibers at a membrane level. Moreover, considering TMEM16A channels as an early target of asbestos or vitreous fiber damage at the cellular level and their expression in *Xenopus* oocytes, the use of the latter model would merit consideration as a promising powerful tool for the safety screening of asbestos replacement vitreous silicate fibers.

## 4. Materials and Methods

### 4.1. Isolation of Xenopus Oocytes

The protocol for *Xenopus* oocyte isolation was approved by the *Organismo Preposto al Benessere degli Animali* (*OPBA*, University of Trieste), the Italian Ministry of Health under authorization number 719/2021-PR, and international laws and policies (European Economic Community, Council Directive 63/2010 Italian D.L. 26/2014). Briefly, sexually mature frog females were anesthetized by immersion in a 0.17% tricaine methane sulfonate (MS-222) solution and ice, for about 15 min. The ovarian pieces were aseptically removed, through an incision of less than 1.5 cm in length. Follicle cells surrounding the oocytes were mechanically removed with fine-tipped forceps, and treated enzymatically for 35 min with collagenase type I (0.5 mg/mL, SIGMA) at room temperature. Oocytes were incubated at 16 °C in Barth’s solution (NaCl 88 mM, KCl 1 mM, Ca(NO_3_)_2_ 0.33 mM, CaCl_2_ 0.41 mM, MgSO_4_ 0.80 mM, NaHCO_3_ 2.4 mM, and HEPES 10 mM, adjusted to pH 7.4 with NaOH), with addition of gentamicin (50 µg/mL). 

### 4.2. Electrophysiological Recordings

To allow the healing of the damaged membrane due to the collagenase treatment, the recordings were performed using a two-electrode voltage clamp (TEVC) technique, 24 h after oocyte isolation. Ten to fifteen oocytes (stage VI) in 1.5 mL Eppendorf tubes were incubated in 1 mL of Ringer solution (NaCl 115 mM, KCl 2 mM, CaCl_2_ 1.8 mM, amd HEPES 5 mM, adjusted to pH 7.4 with NaOH) or Ca11 solution (NaCl 95.6 mM, KCl 2 mM, CaCl_2_ 11 mM, and HEPES 5 mM; pH 7.4), alone (Ctrl) or in the presence of fibers (crocidolite or FAV173), (Fe^3+^ + H_2_O_2_) (FeCl_3_, 400 μM, pH 7.4 + 1 mM H_2_O_2_), with or without cytochalasin D [18,19], and continuously mixed for about 5–35 min, 60 min, or 120 min (wheel, 7 revolutions/min), depending on the aims. The glass intracellular recording micropipettes were filled with KCl (3M), with a tip resistance of 0.5–2 MΩ, and connected to a recording preamplifier (Oocyte Clamp OC-725C). During the recordings, the cells were continuously superfused with the normal bath solution (Ringer) or with the Ca11 solution, depending on test conditions. The resting membrane potential (RP) values were recorded 3–5 min after impalement, and the membrane input resistance (R_m_) values were estimated from the 1/slope of I–V relationships measured by voltage clamping the membrane from −70 to −40 mV, to likely evoke leak currents (with no rectification). The I–V relationships were obtained by measuring the currents at different voltages, from −80 to +40 mV (3 s), 10 mV steps, at a holding potential of −40 mV. To reduce the donor variability, the results were usually normalized with respect to control (Ctrl) of the same donor, unless the comparison was made among cells of the same batch (donor). 

Data acquisition and analyses were performed by WinWCP version 4.1.2 Strathclyde Electrophysiology software, kindly provided by Dr. John Dempster (Glasgow, UK). 

### 4.3. Asbestos and FAV173 Fibers 

Analytical Standard UICC samples of crocidolite (Croc) were purchased from SPI-CHEM, West Chester, PA, USA. The reference batch of the standard sample was crocidolite South Africa (CAS 02704-AB). The fibers spanned from 0.5 to 100 μm in length and from 0.1 to 1.2 μm in width. The Rock Wool FAV173, (CAS-650-016-00-2), was provided by ANALITICA S.A.S. di Francesco Dellisanti.

Dry asbestos and FAV173 fibers were handled in a hood Multihazard Glovebox to prevent inhalation at Azienda Sanitaria Universitaria Giuliano-Isontina (ASUGI). The Croc was re-suspended in phosphate-buffered saline (PBS) at a final concentration of 10 mg/mL, and stored at 4 °C until use. The sheets of FAV173 were ground in a ceramic mortar for 10 min and resuspended in Ringer’s solution at a final concentration of 10 mg/mL. To avoid large fiber aggregates, the solutions were left to sediment for 2 min beforehand and aliquots resuspended in 1 mL of Ringer’s Ca11 solution, at the final concentration.

### 4.4. Scanning and Transmission Electron Microscopy 

Scanning electron microscopy (SEM) was performed using a LEO 1540XB microscope (Carl Zeiss, Oberkochen, Germany). Oocytes were initially fixed with 2.5% glutaraldehyde (Serva, Heidelberg, Germany) dissolved in Ringer or Ca11 solution at room temperature for 20 min, followed by rinsing. Afterwards, the samples were dehydrated through a graded ethanol series (30%, 50%, 60%, 90%, and 100%) and subjected to critical point drying (CPD) in 100% ethanol using a Tousimis, Samdri 780-A instrument (Rockville, MD, USA). 

### 4.5. Chemicals

Ani9, Cytochalasin D, Tricaine methane sulfonate MS-222, and Collagenase type I were purchased from Sigma-Aldrich (St. Louis, MO, USA), Glutaraldehyde from Serva, Heidelberg, Germany 

### 4.6. Statistical Analysis 

Prism 3.0 and Origin 2021 were used for statistical analysis. Statistical significance for comparison between two different groups was established using a Student’s *t*-test or ANOVA for multiple comparisons (more details of the analysis are specified in the figure’s legends). All values are expressed as mean ± SEM (*p*-value: * *p* < 0.05, ** *p* < 0.01, *** *p* < 0.001). 

## Data Availability

The data presented in this study are available on request from the corresponding author.

## Supplementary Materials

The following supporting information can be downloaded at: https://www.mdpi.com/article/10.3390/ijms25168661/s1.
