# Supplementary material for: Effect of Synthetic Vitreous Fiber Exposure on TMEM16A Channels in a Xenopus laevis Oocyte Model
Source: Int J Mol Sci. 2024 Aug 8;25(16):8661. doi: 10.3390/ijms25168661 (PMC11354525; doi:10.3390/ijms25168661)
Supplement: Supplementary file 1 [file ijms-25-08661-s001.zip › ijms-3115459-supplementary.pdf]

SI information

Table S1. The comparison of the chemical composition of the rock wool (FAV173) and Crocidolite standard UICC (Croc) collected by SEM-EDS analysis (Analitica S.a.s di Francesco Dellisanti & C, BO, Italy) expressed as a weight percentage.

w/w %	SiO_2	TiO_2	Al_2O_3	Fe_2O_3	MnO	CaO	MgO	Na_2O	K_2O	Ba	H_2O
FAV173	39.2	2.7	17.8	10.7	0.7	16.5	7.9	2.9	1.8	-	-
Croc UICC	54.6	-	-	26.5			6.8	10.2	-	-	1.9

Materials and Methods

Human lung cancer A549 cells, purchased from Sigma Aldrich, were cultured in DMEM supplemented with 10% fetal bovine serum (FBS) and antibiotics (penicillin, 100 U/mL; streptomycin 100 μg/mL) at 37°C in a humidified atmosphere of 5% CO_2. The cells were grown to approximately 80% confluence in 35 mm Petri plates. The patch clamp recordings were performed in whole-cell configuration using borosilicate glass pipettes (3-5 MΩ tip resistance). The A596 cells were kept in a bath solution containing (mM): NaCl 140, KCl 5, $MgCl_2$ 5, glucose 10, HEPES-NaOH 10, pH 7.4. The pipettes were filled with the following solution (mM): KCl 120, $CaCl_2$ 1, $MgCl_2$ 2, EGTA 11, HEPES-KOH, pH 7.3. The evoked membrane currents under voltage clamp were measured by holding the membrane potential at -40 mV, and stepping the voltages from -100 mV to +90 mV (10 mV intervals, 500 ms). The currents and the resting membrane potential were measured using an Axopatch 200A. The signals were digitized at 10 kHz and low-pass filtered at 2 kHz (interface DigiData 1321A). Leakage currents were digitally subtracted using a P/4 protocol. The analysis was performed using Clampfit 10.2 (Axon Instrument).

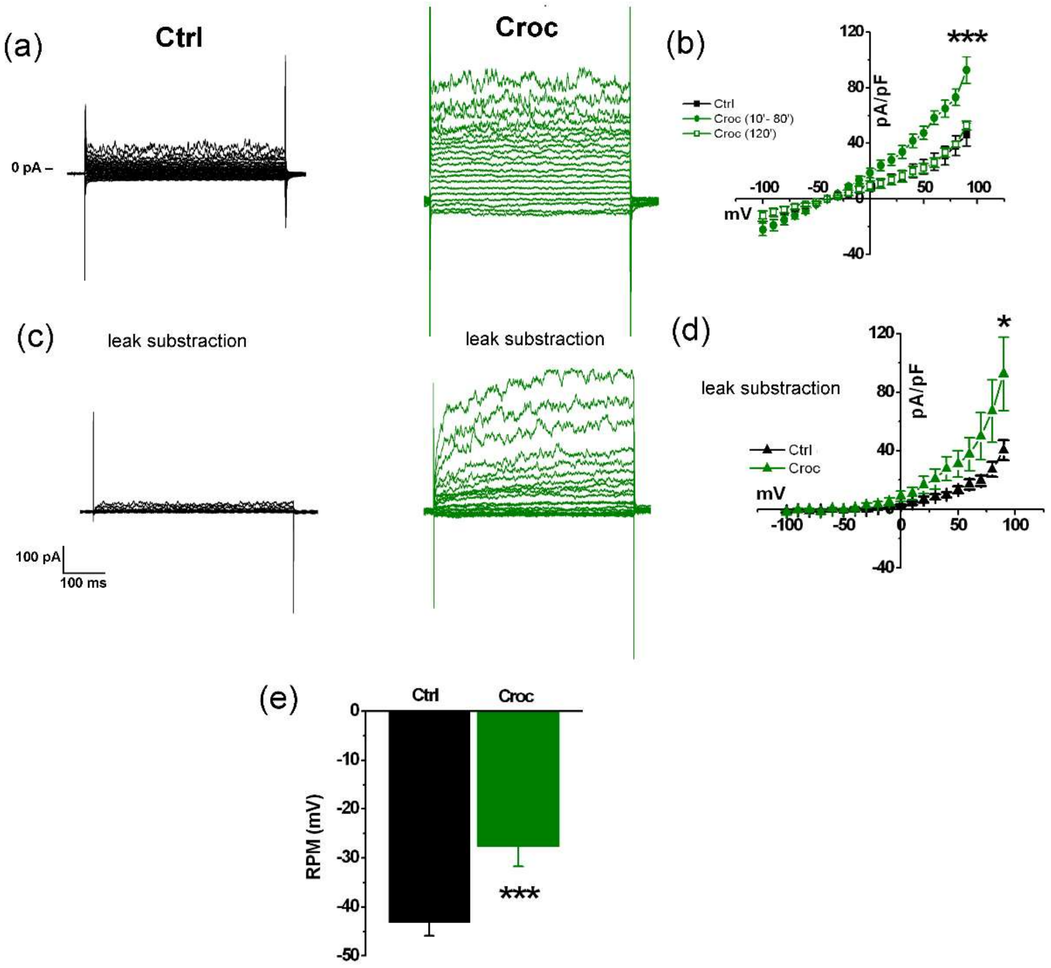

Figure S1 Croc-mediated currents in A549 cells.

In **(a)** is an example of membrane currents evoked by stepping the voltage of the membrane from -100 mV to + 90 mV, recorded in whole-cell configuration, in Ctrl and Croc-treated cells (Croc: 15 µg/ml). In **(b)** the I-V relationships of Ctrl (n = 11) and Croc-treated cells (n = 8, 10 - 80 min of fiber incubation). Note that the Croc effect reversed within 120 min of treatment (n = 5). In **(c)** current traces after leak subtraction, and in **(d)** the I-V relationships of the currents without leak, where the outward rectification similar to that of the TMEM16A channels is clearly visible. The Croc treatment significantly increased both current amplitudes (*e.g.* at + 90 mV, ***$p < 0.001$ with leak, *$p < 0.05$ without leak vs Ctrl), and depolarized the resting membrane potential of the cells, as shown in **(e)** (Ctrl: -43.23 ± 2.67 mV, n = 22, Croc: -27.54 ± 4.19 mV, n = 13). Unpaired t-test.
